# A Case of a Patient With Cannabis Hyperemesis Syndrome Along With Recurrent Nephrolithiasis

**DOI:** 10.7759/cureus.37182

**Published:** 2023-04-05

**Authors:** Maitree Patel, Rajalakshmi Sathiya Narayanan, Appala S Peela

**Affiliations:** 1 Medicine, Boston Children's Hospital, Boston, USA; 2 Internal Medicine, Stanley Medical College, Chennai, IND; 3 Family Medicine, University of North Carolina (UNC) Health Southeastern, Lumberton, USA

**Keywords:** renal calculi, opioid misuse, cannabinoid receptors, marijuana usage, nephrolithiasis, cannabis hyperemesis syndrome

## Abstract

Cannabis hyperemesis syndrome (CHS) is a condition characterized by cyclic vomiting and abdominal pain in chronic cannabis users. It is caused by long-term cannabis use and is often misdiagnosed or unrecognized. CHS can lead to dehydration, electrolyte imbalances, and renal failure, exacerbating the risk of nephrolithiasis or kidney stones. Nephrolithiasis is a common urologic condition characterized by the formation of solid stones in the kidneys, ureters, or bladder. The association between CHS and nephrolithiasis is still unclear and requires further investigation. However, it is suggested that CHS may increase the risk of nephrolithiasis due to dehydration and electrolyte imbalances. Therefore, healthcare professionals should be aware of the potential complications of CHS and monitor patients for kidney stones, especially in chronic cannabis users. We report a case of a 28-year-old American-Indian male with a history of daily marijuana use, presented with recurrent renal stones and acute colicky pain.

## Introduction

Nephrolithiasis, also known as renal stones or kidney stones, affects approximately 12% of the world's population, with calcium stones comprising about 80% of all kidney stones. According to the National Health and Nutrition Exam Survey (NHANES), from 2007 to 2010, the prevalence of nephrolithiasis in the United States population was 8.8%, with 10.6% of males and 7.1% of females affected. Kidney stone growth begins with the formation of crystals within supersaturated urine. The crystals adhere to the urothelium to create a nidus, resulting in subsequent stone growth. The calcium oxalate stones develop on calcium phosphate crystals, specifically Randall's plaques, on the renal papillary surfaces that eventually erode the urothelium to form a nucleus for calcium oxalate deposition. Despite no prevalence for age, sex, and race, there is an increased frequency of kidney stones among men aged 20-49. Diabetes, obesity, and hypertension are known risk factors for nephrolithiasis. However, few studies have reported individuals with recurrent nephrolithiasis with cannabis hyperemesis syndrome (CHS) [1]. The following case scenario illustrates the hospital course of a patient diagnosed with hydroureteronephrosis. This case report aims to contribute to the expanding scientific literature and outline the management of nephrolithiasis and the prevention of its recurrence.

## Case presentation

History

A 28-year-old American-Indian male with a past medical history of CHS, marijuana abuse, and tobacco abuse presented to the ED with severe right-sided flank pain associated with nausea and vomiting for one day. The pain was insidious in onset and constant; however, pain improved with the one-liter normal saline bolus, Dilaudid, Tamsulosin, and Ceftriaxone. The patient also reported darker urine, which he associated with dehydration from vomiting episodes. He had a history of kidney stones that had previously passed without problems. His father also had a history of kidney stones.

Physical examination

The patient's vitals were unremarkable (Table 1). Notable physical exam findings include right lower quadrant abdominal tenderness to palpation but no right or left costovertebral angle (CVA) tenderness.

**Table 1:** Vitals chart. °F: Fahrenheit scale; °C: Celsius; mmHg: Millimetre of mercury; bpm: Beats per minute; SpO2: Saturation of peripheral oxygen.

Vitals	Inference	Reference range
Temperature	97.6 degrees F	97°F (36.1°C) to 99°F (37.2°C)
Blood pressure (BP)	111/57 mm Hg	Systolic: less than 120 mm Hg; Diastolic: less than 80 mm Hg
Heart rate	53 bpm	60-100 beats per minute
Oxygen Saturation (SpO2)	92% (on room air)	95% or higher

Laboratory findings

The blood and urine investigation reports are provided in Table 2.

**Table 2:** Laboratory results. mg/dL: Milligrams per deciliter; mmol/L: Millimoles per litre; pH: Power of hydrogen; L: Litre.

Test name	Result	Biological reference intervals
Blood urea nitrogen (BUN)	13	6 to 24 mg/dL (2.1 to 8.5 mmol/L )
Creatinine	1	For adult men: 0.74 to 1.35 mg/dL (65.4 to 119.3 micromoles/L); For adult women: 0.59 to 1.04 mg/dL (52.2 to 91.9 micromoles/L)
WBC count	18.7*	4,500 to 11,000 WBCs per microliter (4.5 to 11.0 × 109/L)
* URINE ANALYSIS*		
Appearance	Turbid orange	Clear-to-pale yellow
Specific gravity	1.031*	1.005 to 1.030
pH	8.0	4.6 to 8.0
Blood cells	3+	Four RBCs per high power field (RBC/HPF) or less
Protein	1+	Negative
Ketones	1+	Negative
Leukocyte esterase	Trace	Negative
Nitrites	Nil	Negative
Glucose	Nil	Negative
Bilirubin	Nil	0.2-1.0
*URINE MICROSCOPY*		
RBCs	>100*	<5
WBCs	26-50*	<5
Bacteria	Few with significant mucus*	Absent

**Figure 1:**
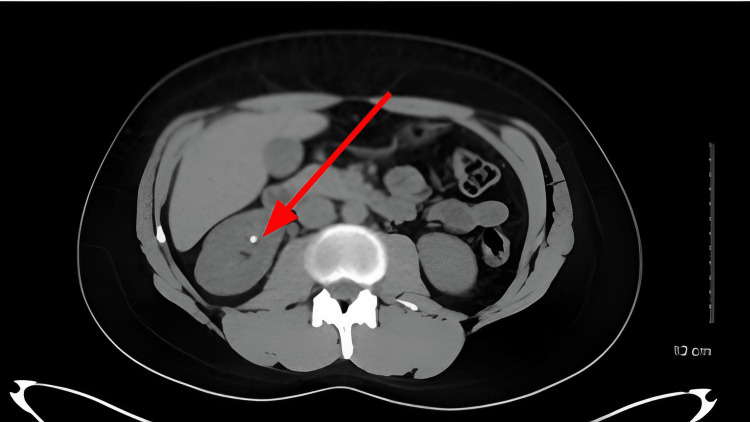
CT image with left renal stone.

 The CT scan findings are shown in Figure 1.

 The crystallographic analysis of urinary calculi is provided in Tables 3-4.

**Table 3:** Crystallographic analysis of urinary calculi.

Specimen	Single, crenated, ovoidal calculus
Weight	34.5 milligrams
Analysis report	
Calculus is composed of an aggregate of octahedral crystals of calcium oxalate dihydrate, with interspatial laminae of columnar monoclinic needles of calcium oxalate monohydrate. The composition of the apparent nidus and inter-crystalline deposits is microcrystalline hydroxyl and carbonate apatites. A protein matrix is demonstrated.	

**Table 4:** Principal stone components.

Chemical name	Approximate percentage	Nidus	Mineralogical name	Formula
Calcium Oxalate Monohydrate	20%		Whewellite	Ca C_2_O_4_·H_2_O
Calcium Oxalate Dihydrate	60%		Weddellite	CaC_2_O_4_·2H_2_O
Calcium Phosphate (Carbonate form)	3%	*	Carbonate apatite	Ca_5_(PO_4_,CO_3_)3(OH,F)
Calcium Phosphate (Hydroxyl form)	15%	*		Ca₁₀(PO₄)₆(OH)₂
Protein	2%			

## Discussion

Long-term use of cannabinoids can cause CHS, a paradoxical disorder characterized by cyclical intractable vomiting, nausea, and abdominal pain. It is often difficult to diagnose, and the epidemiological data, including the prevalence of CHS, remain largely unknown [1]. A case series study by the Mayo Clinic consisting of 98 patients describes the clinical manifestations in three phases and also finds that the pain experienced by patients is opposed to the traditional description of colicky pain [2]. Compulsive hot water showering after 7-10 intensifying episodes of vomiting are typical of CHS. Cannabinoids are used for pain management in chronic conditions and renal stone patients. Increased use of cannabinoids for recreational purposes following their legalization in many states may increase the incidence of CHS [3]. Information on the effects of synthetic cannabinoids on the development and progression of kidney disease is very limited. Their action on the endocannabinoid system, including the CB1 and CB2 receptors in kidneys and other organs, can be both beneficial and harmful [4]. Kidney stones develop when crystal-forming agents, including calcium, oxalate, and uric acid, are not diluted enough in the urine. There are four types of renal stones: calcium, struvite, uric acid, and cystine [5]. A study by Khan SR et al. shows that stone formation is highly prevalent, with a recurrence rate of up to 50% within the first five years of the initial renal stone episode. Medical conditions such as obesity, hypertension, diabetes, and other metabolic syndromes are considered risk factors for stone formation leading to hypertension, chronic kidney failure (CKD), and end-stage renal disease (ESRD). The case described shows that CHS should always be considered in a chronic, heavy cannabis consumption setting with multiple episodes of vomiting and abdominal pain. This patient's formation of recurrent renal stones can be attributed to chronic dehydration and metabolic dysfunction, as explained by dark urine and family history in the case description. Furthermore, this is the first case of CHS in a young adult witnessing recurrent renal stones and also provides witness to the potential role of cannabinoids in nephrolithiasis. Treatment of CHS is an IV fluid substitution, symptomatic management, and rehabilitation. Resistance to antiemetics like ondansetron and meta loperamide is noted. Therefore, treatment options should include benzodiazepines, antiepileptics (levetiracetam), or antipsychotics (haloperidol). Opioid prescription for pain management may worsen nausea and vomiting; hence it is not recommended for CHS. Capsaicin cream is demonstrated to relieve pain and vomiting significantly [6]. Prevention of recurrent renal stones in CHS is aimed at abstinence and adequate hydration. 

Future directions and research recommendations

Stimulation of CB1 and CB2 receptors and the activation of the endocannabinoid system in kidneys may significantly impact renal function, as explained in mice studies and diabetes patients [4]. It is important to study the physiological role of these receptors and ligands to elucidate their potential role in promoting renal stone formation. Large cohort studies will help identify such events. Randomized control trials would be unethical, considering the consequences of cannabis exposure.

## Conclusions

The increasing trend of cannabinoid use since legalization has led to increasing admissions to emergency care. While consumed to affect the central nervous system, the side effects on the kidney can be deleterious. Medical cannabinoids are expected to possess more pharmacologically beneficial properties, but their mechanism of action on the endocannabinoid system is not fully discovered. An observational study in young adults demonstrated no meaningful translation in kidney function concerning marijuana use. Our case provides contrasting evidence for the study. Hence, further research is insisted on observing renal parameters in young adults showing hyperemesis symptoms.
